# Elucidation of short linear motif-based interactions of the MIT and rhodanese domains of the ubiquitin-specific protease 8

**DOI:** 10.1186/s13062-025-00638-7

**Published:** 2025-05-06

**Authors:** Aimiliani Konstantinou, Julia K. Varga, Alicia Córdova-Pérez, Leandro Simonetti, Lidia Gomez-Lucas, Ora Schueler-Furman, Norman E. Davey, Yogesh Kulathu, Ylva Ivarsson

**Affiliations:** 1 Department of Chemistry-BMC, Uppsala University, Box 576, Uppsala, 751 23 Sweden; 2 Department of Microbiology and Molecular Genetics, Institute for Biomedical Research IMRIC, Faculty of Medicine, Hebrew University of Jerusalem, Jerusalem, Israel; 3 MRC Protein Phosphorylation & Ubiquitylation Unit, School of Life Sciences, University of Dundee, Dundee, UK; 4 Division of Cancer Biology, The Institute of Cancer Research (ICR), London, UK

**Keywords:** USP8, MIT domain, Rhodanese domain, SLiMs, Peptide-phage display, Deep mutational scanning

## Abstract

Ubiquitin-specific protease 8 (USP8) is a deubiquitinating enzyme with essential functions in protein trafficking and stability. It is a multidomain protein, with an N-terminal MIT (microtubule interacting and trafficking) domain, followed by a non-catalytic rhodanese (Rhod) domain, a long intrinsically disordered region, and a C-terminal catalytic domain. The N-terminal MIT domain of USP8 is known to mediate protein-protein interactions through binding to short linear motifs. The non-catalytic Rhod domain is also involved in protein-protein interactions, however detailed insights into these interactions remain limited. In this study we explore the short linear motif-based interactions of the MIT and Rhod domains of USP8 using a combination of proteomic peptide-phage display, peptide arrays and deep mutational scanning. We show that the MIT domain can bind ligands with a general [DE][LIF]x{2,3}R[FYIL]xxL[LV] consensus motif. We uncover that the rhodanese domain of USP8 is a peptide-binding domain, and define two distinct binding motifs (Rx[LI]xGxxxPxxL and G[LV][DE][IM]WExKxxxLxE) for this domain by deep mutational scanning of two different peptide ligands. Using the motif information, we predict binding sites within known USP8 interactors and substrates and validate interactions through peptide array analysis. Our findings demonstrate that both the USP8 MIT and rhodanese domains are peptide-binding domains that can be bound by degenerate and distinct binding motifs. The detailed information on the peptide binding preference of the two N-terminal domains of USP8 provide novel insights into the molecular recognition events that underlie the function of this essential deubiquitinating enzyme.

**Supplementary Information:**

The online version contains supplementary material available at 10.1186/s13062-025-00638-7.

## Introduction

The ubiquitin carboxyl-terminal hydrolase 8 (USP8; also known as Ubiquitin isopeptidase Y, UBPY), is an essential deubiquitinating enzyme (DUB). Functionally, USP8 plays a crucial role in regulating membrane trafficking pathways, such as endosomal sorting. Additionally, it is involved in deubiquitination of membrane proteins, thereby regulating their stability [1, 2]. USP8 also regulates the stability of the ESCRT-0 complex proteins HRS (hepatocyte growth factor-regulated tyrosine kinase substrate), STAM1 and STAM2 (signal transducing adapter molecule 1 and 2) [3]. USP8 has further been reported to have nuclear functions, where it is involved in the regulation of DNA-damage response and maintenance of chromatin stability by deubiquitinating the protein microcephalin1 (MCPH1, also called BRIT1) [4]. Deregulation of USP8 is linked to several diseases, such as Cushing’s disease and Parkinson’s disease [5–9]. Somatic mutations in the USP8 gene are implicated in endocrine tumors and other types of cancer [10, 11]. Due to its involvement in multiple diseases, USP8 has attracted attention in therapeutic development [12, 13].

**Fig. 1:**
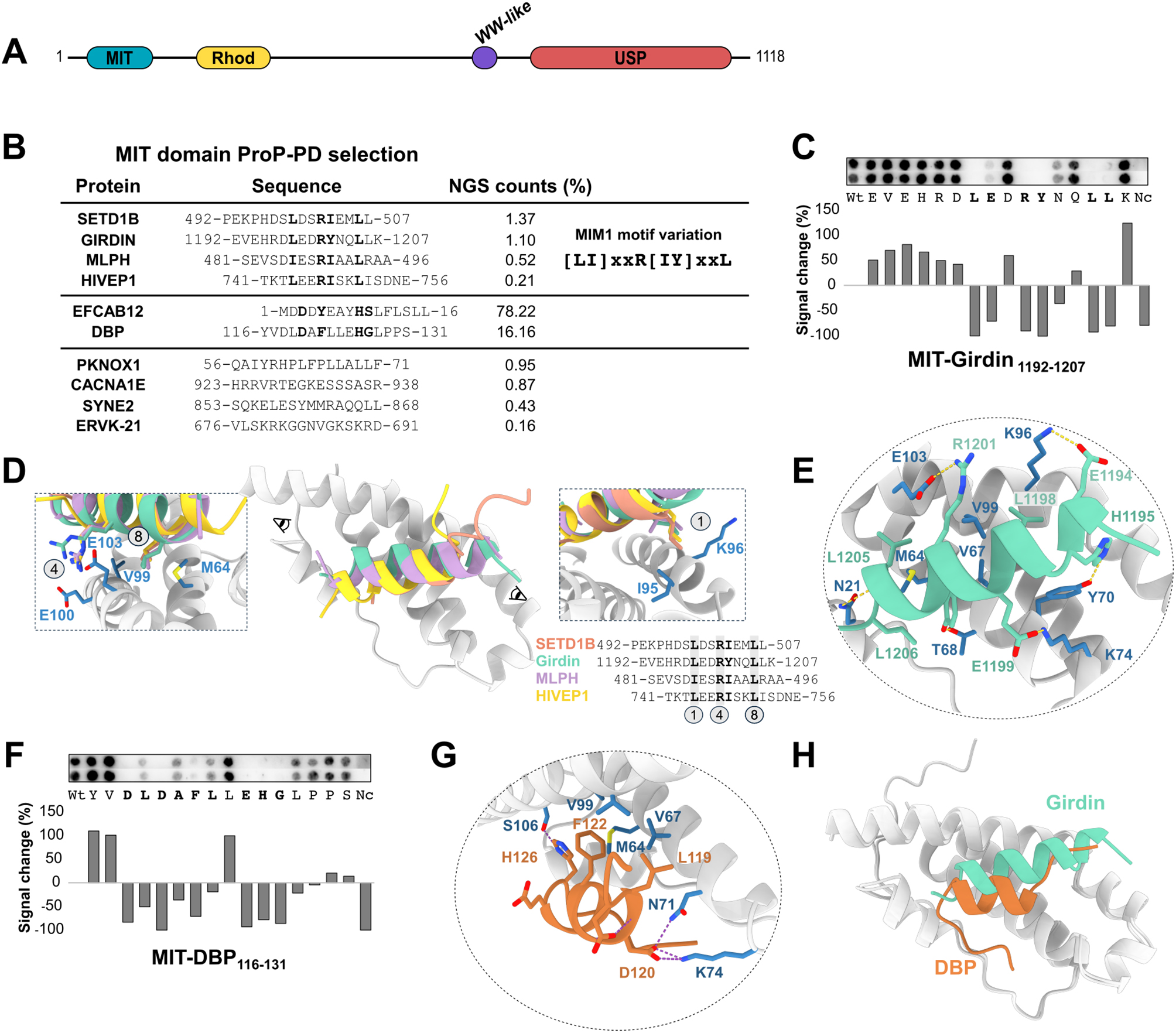
Peptide binding of the USP8 MIT domain. (**A**) Schematic representation of USP8 domain organization: N-terminal MIT domain (MIT), rhodanese-like domain (Rhod), WW-like domain (WW-like), C-terminal USP catalytic domain (USP). (**B**) USP8 MIT peptide-ligands identified through ProP-PD selections. The percent of NGS counts associated with each peptide is indicated. The MIM1-like motif found in four peptides is highlighted in bold, and motif is indicated to the right for comparison. (**C**) SPOT array alanine scanning of the Girdin_1192 − 1207_ peptide binding to the MIT domain. Amino acid residues disrupting binding when mutated to alanine are shown in bold. Signal intensities were normalized to the wild-type (Wt) and displayed as average percent signal change; Nc: negative control (scrambled sequence). (**D**) AF3 models of superimposed MIM1-like motif containing peptides binding to the USP8 MIT domain. The peptide sequence alignment depicts motif residues in bold on grey background, and details of the docking of the key motif positions are shown by insets to the right (1) and left (4 and 8). (**E**) AF3 model of the Girdin_1192 − 1207_ peptide (cyan) binding to the MIT domain. USP8 MIT amino acid residues interacting with the peptide are shown in blue. (**F**) SPOT array alanine scanning of DBP_116 − 131_ peptide binding to the MIT domain. Amino acid residues disrupting binding when mutated to alanine (or glycine in case of wild type alanine) are shown in bold. Signal intensities were treated as describe for panel (**C**). (**G**) AF3 model of DBP_116 − 131_ peptide (orange) binding to the MIT domain. USP8 MIT domain amino acid residues interacting with the peptide are shown in blue. (**H**) AF3 superimposed models of Girdin_1192 − 1207_ peptide (cyan) and DBP_116 − 131_ peptide (orange) binding to the same binding pocket as the MIM1-like containing peptides in (**D**)

Structurally, USP8 consists of several key domains (Fig. 1A; Supplementary Fig. S1): an N-terminal microtubule interacting and trafficking (MIT) domain, a fifty amino acid intrinsically disordered region (IDR) followed by catalytically inactive rhodanese (Rhod) domain, a long IDR that links to an autoinhibitory WW-like domain [14], which docks to a C-terminal catalytic domain (USP) [15] that catalyses the removal of ubiquitin from the substrate. The N-terminal MIT domain of USP8 is a three-helix bundle domain and has been shown to be essential for recruitment to the endosomes through binding to ESCRT-III proteins. This endosomal localization signal is necessary for epidermal growth factor receptor (EGFR) and cytotoxic T-lymphocyte associated protein 4 (CTLA4) receptor degradation [1, 16]. The USP8 MIT domain has previously been shown to be involved in short linear motif (SLiM)-based interactions. In particular, it has been reported to bind to MIT interacting motif 1 (MIM1) containing ESCRT-III proteins (e.g. CHMP1B, CHMP2A/B and CHMP4A/B) [1, 17]. The MIM1 motif is characterised by a [DE]xxLxxRLxxL[KR] (where x is any amino acid, except proline) consensus motif, as reported previously for the MIT domain of Vps4 [18, 19]. The USP8 MIT domain also has been proposed to dimerise [15], although a recent study showed full-length USP8 in a monomeric state in solution [20]. USP8 is the only human DUB known to have a rhodanese-like domain (Rhod domain). The Rhod domain consists of five β-strands surrounded by α-helices. In contrast to catalytically active Rhod domains found in human sulfurtransferase and phosphatases [21, 22], the USP8 Rhod domain lacks a conserved active site cysteine. The USP8 Rhod domain has been implicated in protein-protein interactions, with a loop region of the rhodanese domain being bound to the C-terminal region of NRDP1 E3 ligase [15, 23]. Beyond that, its function remains unclear. Finally, the USP8 IDR contains a phospho-dependent SLiM, which is recognized by 14-3-3 proteins and is a mutational hot spot associated with Cushing’s disease [5, 24]. The IDR also harbours three non-canonical SH3 binding motifs (RxxK) [25, 26], mediating binding to the SH3 domains of STAM1/2, which facilitate the formation of the endosomal sorting complex 0 (ESCRT-0) [25–27].

In this study we explore the potential of the USP8 MIT domain and Rhod domain to bind to short linear peptide motifs using a proteomic peptide-phage display (ProP-PD) library displaying about 1 million 16 amino acid long peptides, tiling the IDRs of the human proteome [28]. We identify novel ligands of the USP8 MIT, and pinpoint variations of the previously described MIM1 motif. We further uncover that the USP8 Rhodanese domain is a peptide-binding domain. We dissect the peptide-binding specificity determinants of the USP8 MIT and Rhod domains using a deep mutational scanning (DMS) approach and predict binding sites in previously reported USP8 interaction partners and substrates. Our study provides novel insights into the SLiM-based interactions of USP8 MIT and Rhod domains, broadening the mechanisms of USP8 molecular recognition.

## Results

### Identification and characterisation of USP8 MIT ligands

We explored the peptide-binding of the USP8 MIT domain by using the purified GST-tagged MIT domain as bait in proteomic peptide-phage display (ProP-PD) selections and a library designed to display the predicted unstructured regions of the human proteome [28]. The peptide-coding regions of the binding-enriched phage pools were analysed by next-generation sequencing (NGS). After filtering for high confidence ligands, ten ligands remained (Fig. 1B; Supplementary Table S1). None of the ten identified protein-protein interactions had been previously reported, based on comparison with the information available in IntAct, HIPPIE and BioGrid [29–31] (February 2025). Of the USP8 interacting proteins found, the D site-binding protein (DBP), which is a clock-controlled transcription factor, appears as an interesting ligand as USP8 has been reported to be involved in controlling the circadian rhythm [32]. Also, the CCDC88A-encoded protein Girdin is an interactor of potential biological relevance as it is a scaffolding protein involved in the EGFR pathway [33, 34] which USP8 is known to regulate [26, 35]. At the motif level, there was no shared consensus motif among the ten peptide ligands. However, by manual inspection we noticed that four of the peptides contained a shared [LI]xxR[IY]xxL sequence, which appears to be a variation of the [DE]xxLxxRLxxL[KR] MIM1 motif described in the Eukaryotic Linear Motif resource (ELM) [19] (Fig. 1B). We performed a SPOT array alanine scanning analysis in which peptides are synthesised on a membrane, and where each amino acid position of the peptide is mutated to alanine (or glycine if alanine is in the wild type peptide) to evaluate the effect of the amino acid residues in binding. We tested two peptides containing the apparent MIM1-like motif, one from Girdin (Girdin_1192 − 1207_) and one from the histone-lysine N-methyltransferase SETD1B (SETD1B_492 − 507_). The alanine scanning analysis revealed that binding to the Girdin_1192 − 1207_ peptide was lost when the residues LExRYxxLL were mutated to alanine (Fig. 1C) confirming a variation of the core of the MIM1 motif and suggesting a contribution of a glutamic acid at the p2 position. The SPOT array alanine scanning of SETD1B_492 − 507_ further confirmed the contributions of the motif residues in binding and the aspartic acid in position p2 of the motif (Supplementary Fig. S2). AlphaFold3 (AF3) modelling [36] of the four peptides with the variant MIM1 motif and the MIT domain docked the peptides with high confidence (ipTM scores: HIVEP1_741 − 756_: 0.87, Girdin_1192 − 1207_: 0.85, MLPH_481 − 496_: 0.82, SETD1B_492 − 507_: 0.75) at the expected MIM1 binding pocket (Fig. 1D). The AF3 model of the Girdin_1192 − 1207_ peptide binding to the MIT domain corroborated the importance of the LExRxxxL motif residues for the interaction (Fig. 1E) and suggests that the arginine in the fourth position (p4) of the motif interacts through electrostatic interactions with E103 in the MIT domain. The model further suggests that the contribution of glutamic acid at the second position of the motif (p2) stems from an interaction with K74 in the MIT domain. Thus, four of the ProP-PD derived USP8 MIT binding peptides contain a shared MIM1 variant motif. However, the USP8 MIT selection was dominated (more than 94% of NGS counts) by two peptides that lack an apparent MIM1 motif. The two peptides were from the EF-hand calcium-binding domain-containing protein 12 (EFCAB12_1 − 16_: MDDDYEAYHSLFLSLL) and from DBP (DBP_116 − 131_: YVDLDAFLLEHGLPPS) (Fig. 1B). Notably, the DBP_116 − 131_ and EFCAB12_1 − 16_ peptides lack arginine residues, which is central to the MIM1 motif. Alanine scanning by peptide SPOT array analysis of the DBP_116 − 131_ peptide revealed the importance of an extended DxDxFxxEHG stretch (Fig. 1F). In comparison to this, the EFCAB12_1 − 16_ peptide shares a potential Dx[YF]xxxH[SG] motif with the DBP_116 − 131_ (Fig. 1B). AF3 modelling of the DBP_116 − 131_ peptide and USP8 MIT domain complex suggested with high confidence (ipTM score 0.84) that the peptide binds as an α-helix (Fig. 1G). Overlaying the model of the MIT-DBP_116 − 131_ complex with the complex of the MIT domain bound to the Girdin_1192 − 1207_ peptide suggested that the two peptides bind to a partially overlapping binding site on the MIT domain, with the DBP_116 − 131_ peptide making a shorter helical structure than the Girdin_1192 − 1207_ peptide (Fig. 1H). The AF3 model of DBP_116 − 131_ peptide supports the interaction of D120 of the DBP_116 − 131_ peptide with the residues K74 and N71 in the MIT domain (Fig. 1G), emphasizing the significance of the aspartic acid at this position. The alanine scanning results together with the AF3 modelling suggests that the USP8 MIT domain can bind MIM1-like motifs and degenerate variants thereof using a partially overlapping binding site. Finally, we note a third group of four peptides that did not conform to the MIM1-like motif or the DBP-type MIT binding motif (Fig. 1B). Of them, two peptides (SYNE2_853 − 868_ and PKNOX1_56 − 71_) where placed in the same binding site as the Girdin and DBP peptides with high confidences by AF3 modelling (ipTM 0.92 and 0.74; Supplementary Fig. S3). Taken together, the experimental results indicate that the USP8 MIT domain can accommodate peptides with diverse amino acid sequences.

### Deep mutational scanning of the USP8 MIT binding motifs

**Fig. 2:**
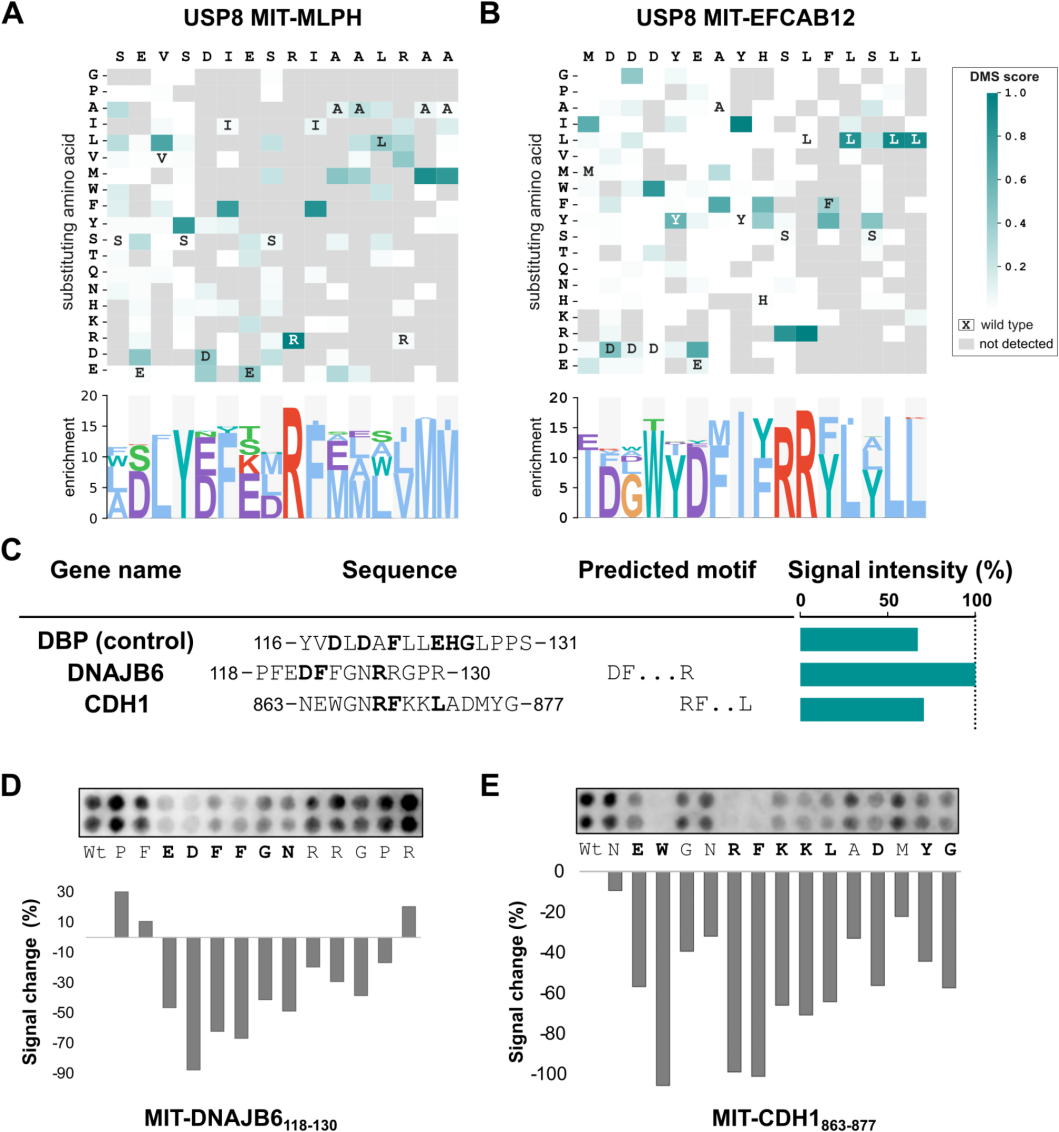
DMS analysis of MIT domain binding peptides and validation of predicted binding peptides from known interactor. (**A**, **B**) Heatmap representation of the PSSMs generated by the peptide-phage display-based DMS analysis of the two indicated peptides. (**C**) SPOT array results of selected peptides from known interactors tested for binding to the MIT domain. The highest scoring USP8 MIT binding peptides based on peptide array analysis of 24 predicted ligands are shown. The amino acid residues of the predicted motifs are indicated in bold. The DBP_116 − 131_ peptide was used a positive control, and the signal intensities were normalized to the highest intensity observed (that is, for DNAJB6) and indicated as percentage of max signal. The SPOT array and additional information can be found in Supplementary Fig. S4 and Supplementary Table S4. (**D**) SPOT array alanine scanning of DNAJB6_118 − 130_ peptide binding to the MIT domain. (**E**) SPOT array alanine scanning of CDH1_863 − 877_ peptide binding to the MIT domain. (**D**,** E**) Signal intensities were normalized to wild type (Wt) and displayed as average percent signal change

To further elucidate the peptide binding determinants of the interactions with USP8 MIT domain we performed a deep mutational scanning (DMS) analysis of two model peptides: one peptide from melanophilin (MLPH_481 − 496_: SEVSD**IE**S**RI**AA**L**RAA) which served as a representative of the MIM1-like motif and the EFCAB12 peptide (EFCAB12_1 − 16_: MD**D**D**Y**EAY**HS**LFLSLL), which shares some sequence similarity with the DBP_116 − 131_ peptide. We designed a library of peptides in which each position of the two parental peptides were mutated to all other amino acids (except cysteine) following a classic saturation mutagenesis approach (Supplementary Table S2A). The peptides were displayed on the surface of the M13 phage and used in triplicate selections in biological replicates against the purified bait proteins, following our recently outlined protocol [37]. Position specific scoring matrices (PSSMs) were generated based on the enrichment of the peptides as revealed by the NGS analysis. The DMS analysis resulted in sparse data (Fig. 2A, B; Supplementary Table S3). For both model peptides the analysis resulted in extended motifs with some shared features, roughly described by [DE]Fx{2,3}R[FY]xxLL. Although starting from two clearly distinct parental peptides, the DMS analysis thus converged on a MIM1-like motif. The convergence of the motifs derived by DMS analysis suggests that although the MIT domain may bind peptides with diverse sequences, it has a preference for a MIM1-like motif. Thus, based on the DMS analysis results, the ProP-PD derived peptides and the previous reports on USP8 MIT binding peptides (e.g. CHMP1B: 187-ELSQRLARLR-196) [17, 38], we define a general USP8 MIT domain consensus binding motif as [DE][LIF]x{2,3}R[FYIL]xxL[LV], which for instance is perfectly represented by the motif found in the Girdin_1192 − 1207_ peptide (**DL**Ex**RY**xx**LL**). However, we note that also partial degenerate motif-matches may be sufficient for binding.

To further assess the SLiM-based interactions of the USP8 MIT domain, we designed a peptide array tiling 24 potential binding sites in previously reported USP8 interactors. We used SLiMSearch [39] to search for peptides that contain shorter or longer matches to the motif (Supplementary Table S4A). While most of the tested peptides did not bind (Supplementary Fig. S4; Supplementary Table S4B) two peptides from the chaperone DnaJ homolog subfamily B member 6 (DNAJB6_118 − 130_) and the cell adhesion protein cadherin-1 (CDH1_863 − 877_) resulted in similar or stronger SPOT intensities than observed for the positive control (the DBP_116 − 131_ peptide; Fig. 2C). The DNAJB6_118 − 130_ peptide was predicted as a binder based on a partial DFx{2,3}R motif. The DNAJB6-USP8 interaction is supported by previous GST-pulldown experiments and colocalization of the two proteins in male mouse germ cells [41], and the interaction is thought to be of importance to the protein quality control to yield functional spermatozoa [41]. In the case of the CDH1_863 − 877_ peptide it was predicted as USP8 MIT ligand based on the presence of an RFxxL motif. The previous support of the CDH1-USP8 interaction is based on proximity labelling mass spectrometry [40], and our results thus provide evidence for a binary interaction between the two proteins and define a binding site. To confirm that the motifs used to predict the interactions were of importance for binding we designed an alanine scanning peptide SPOT array analysis of the DNAJB6_118 − 130_ and the CDH1_863 − 877_ peptides. Alanine scanning of DNAJB6_118 − 130_ confirmed the importance of the aspartic acid and the phenylalanine of the predicted motif (DFx{2,3}R) and showed that the mutations of the flanking residues also reduced binding (Fig. 2D). Alanine scanning of the CDH1_863 − 877_ peptide corroborated the RFxxL motif and revealed a longer stretch is contributing to binding, with an upstream tryptophan being critical for the interaction (Fig. 2E). In summary, we find that the USP8 MIT domain binds to a MIM1-like motif that can be expressed by [DE][LIF]x{2,3}R[FYIL]xxL[LV], as well as degenerate variants thereof.

### Characterisation of the USP8 rhodanese domain ligands and binding motifs

**Fig. 3:**
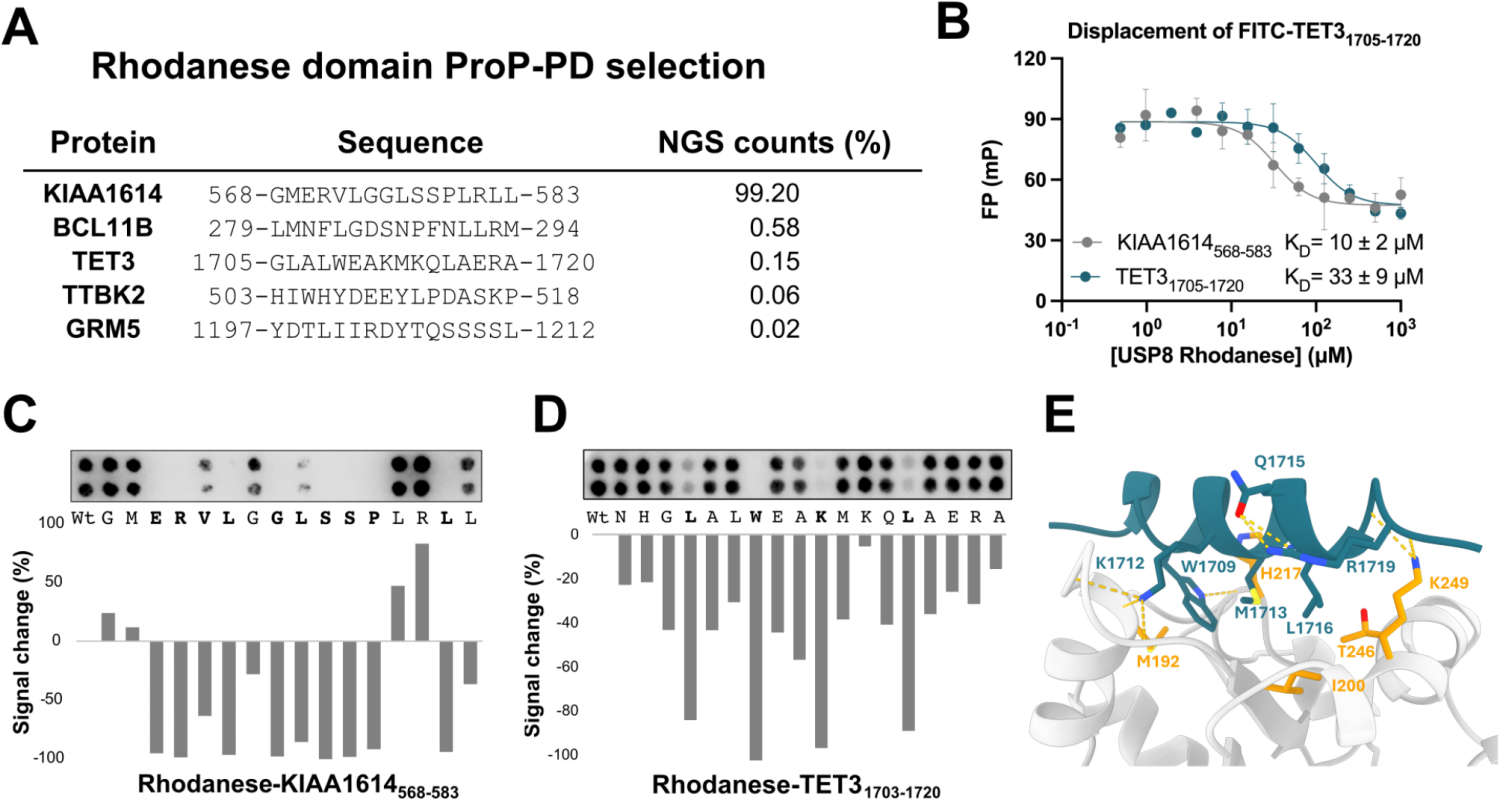
Overview of USP8 Rhod domain binding to ligands identified through ProP-PD selections. (**A**) Peptides of high/medium confidence binding to USP8 Rhodanese domain identified in ProP-PD. (**B**) Fluorescence polarization-monitored experiments of TET3_1705 − 1720_ bound to the USP8 Rhod domain, displaced by the peptide ligands KIAA1614_568 − 583_ and TET3_1705 − 1720_. The data are presented as mean ± SD (*N* = 3 technical triplicates) and the K_D_ values are indicated. (**C**) Peptide SPOT array alanine scanning of KIAA1614_568 − 583_ peptide binding to the Rhod domain. (**D**) Peptide SPOT array alanine scanning of TET3_1705 − 1720_ peptide binding to the Rhod domain. (**C**,** D**) Amino acid residues disrupting binding when mutated to alanine are shown in bold. Signal intensities were normalized to wild type (Wt) and displayed as average percent signal change. (**E**) AF3 model of TET3_1705 − 1720_ peptide (teal) binding to the Rhod domain. Amino acids of the USP8 Rhod domain that interact with the peptide based on the model are shown in yellow

We next evaluated the peptide binding of the Rhod domain by ProP-PD selections. NGS analysis of the binding enriched phage pools identified six peptides, with peptides from the uncharacterised protein KIAA1614_568 − 583_ dominating the selection (Fig. 3A; Supplementary Table S5). The results demonstrate the potential of the USP8 Rhod domain to bind peptide ligands, although none of the USP8 Rhod interacting proteins found have previously been reported to be USP8 interactors based on information compiled in IntAct, HIPPIE and BioGrid [29–31] (February 2025). We validated the interactions of KIAA1614_568 − 583_ and TET3_1705 − 1720_ peptides by a competitive fluorescence polarization (FP) assay, where a preformed complex between USP8 Rhod and a probe peptide (FITC-TET3_1705 − 1720_) was challenged with unlabelled peptides (TET3_1705 − 1720_ or KIAA1614_568 − 583_). The FITC-TET3_1705 − 1720_ probe peptide was efficiently outcompeted by the unlabelled KIAA1614_568 − 583_ and TET3_1705 − 1720_ peptides (Fig. 3B), and the K_D_ values of the interactions were determined to be 10 ± 2 µM for the USP8 Rhod-KIAA1614_568 − 583_ interaction and 33 ± 9 µM for USP8 Rhod-TET3_1705 − 1720_ interaction (Supplementary Table S6). The KIAA1614_568 − 583_ peptide is thus about a three-fold better binder than TET3_1705 − 1720_, consistent with their relative enrichment in the phage selection. To further validate interactions and gain insight into the binding motif we designed an alanine scanning SPOT array using the highly enriched KIAA1614_568 − 583_ peptide, which identified an extended ERVLxGLSSPxxL stretch (Fig. 3C). SPOT array alanine scanning of TET3 peptide (TET3_1703 − 1720_) revealed that an extended LxxWxxKxxxL motif is critical for binding to the Rhod domain (Fig. 3D). AF3 modelling of the USP8 Rhod-TET3_1705 − 1720_ complex resulted in a model with high confidence (ipTM score: 0.88), where the peptide adopts an extended α-helical structure, and the central tryptophan of the peptide docks into a hydrophobic cavity (Fig. 3E). The binding region on the domain is distinct from the active site of catalytically active Rhod domains, as revealed by inspecting the overlay between the models and the crystal structure of the CDC25B catalytic domain (PDB code 1qb0; Supplementary Fig. S5). Despite binding with higher affinity, AF3 modelling of the USP8 Rhod-KIAA1614_568 − 583_ complex resulted in a low confidence model (ipTM score: 0.46). Low confidence models were consistently observed when testing different truncations or extensions of the KIAA1614 peptide, or when testing alternative docking methods (AlphaFold2-multimer [42] Chai-1 [43], PatchMAN [44]). The low confidence of the modelling of the KIAA1614_568 − 583_ peptide to the UPS8 Rhod domain despite a high affinity may relate to a fuzzy mode of interaction as previously described for other cases [45], but this remains to be elucidated by experimental structural approaches.

### Further analysis of USP8 rhodanese domain binding peptides reveals distinct binding motifs

**Fig. 4:**
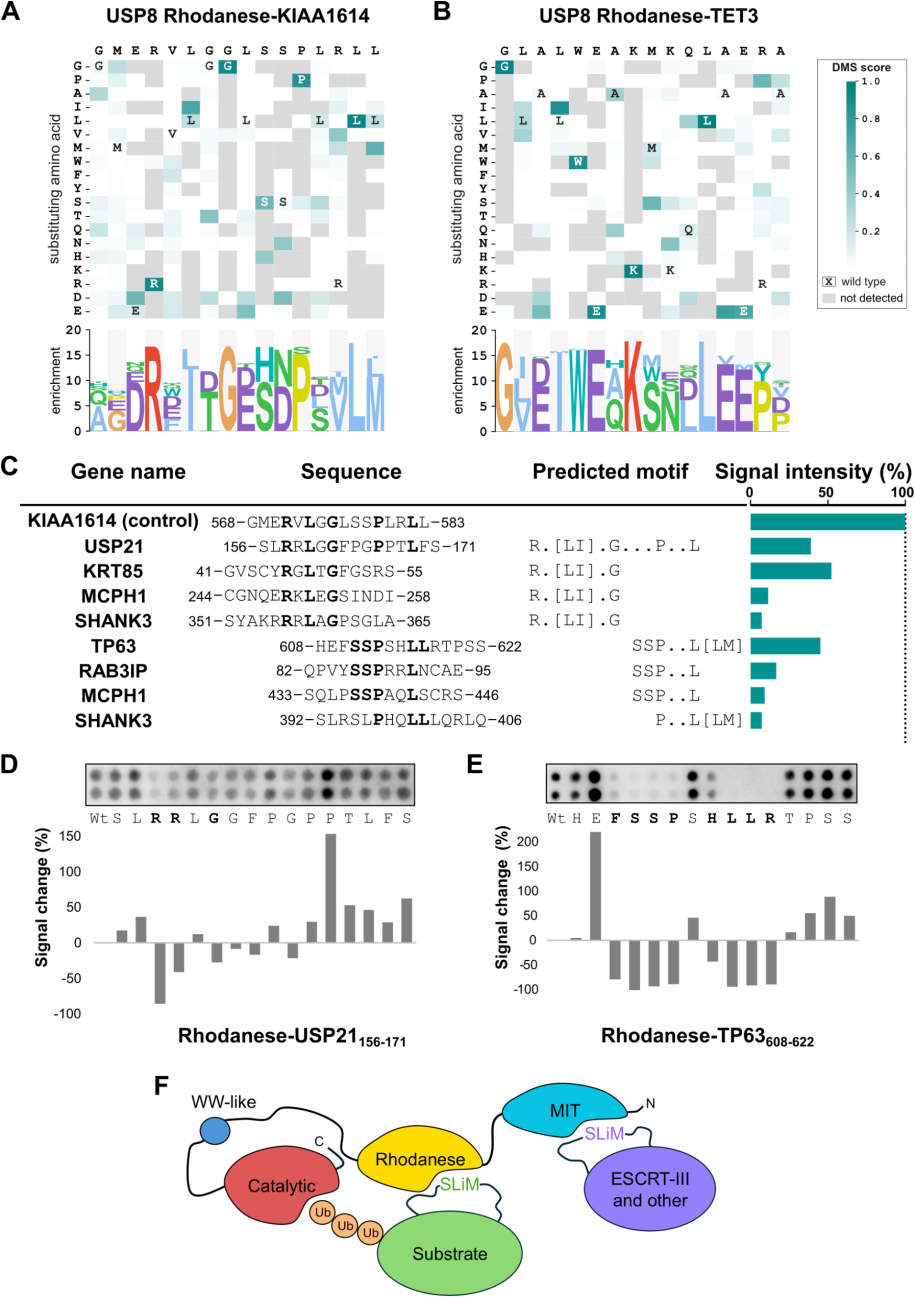
DMS analysis of USP8 Rhod domain binding peptides and predicted peptides from known interactors containing the (partial) motifs generated by DMS. (**A**,** B**) Heatmap representation of PSSMs generated by the peptide-phage display-based DMS analysis of the two indicated peptides. (**C**) SPOT array results of selected peptides from known interactors tested for binding to the Rhod domain. The highest scoring USP8 Rhod binding peptides based on peptide array analysis of 42 predicted ligands are shown. The amino acid residues of the predicted motifs are indicated in bold. The KIAA1614_568 − 583_ peptide was used a positive control, and the signal intensities were normalized to the higher intensity (positive control) and indicated as percentage. The SPOT array and additional information can be found in Supplementary Fig. S6 and Supplementary Table S8. (**D**) Peptide SPOT array alanine scanning of USP21_156 − 171_ peptide binding to the MIT domain. (**E**) Peptide SPOT array alanine scanning of TP63_608 − 622_ peptide binding to the MIT domain. (**D**,** E**) Signal intensities were normalized to wild type (Wt) and displayed as average percent signal change. (**F**) Schematic model of how SLiM-based interactions of the Rhod domain may contribute to substrate targeting of USP8, while the MIT domain interacts with motifs found in ESCRT-III proteins (e.g. CHMPs) and other proteins thereby contributing to the targeting of the protein to different cellular localizations (e.g. endosomes) and interaction partners

To gain additional insight into the USP8 Rhod peptide binding we performed a peptide-phage display-based DMS analysis of two peptides, KIAA1614_568 − 583_, and TET3_1705 − 1720_ (Supplementary Table S2, Supplementary Table S7). In this case, the DMS analysis resulted in two distinct motifs. The KIAA1614_568 − 583_ DMS analysis generated an extended Rx[LI]xGxxxPxxL[LM] motif (Fig. 4A), which is consistent with the E**R**x**L**x**G**xSS**P**xx**L** motif determined by SPOT alanine scanning (Fig. 3C). The DMS analysis of the TET3_1705 − 1720_ peptide resulted instead in a distinct G[LV][DE][IM]WExKxxxLxE motif (Fig. 4B) which resembles the **GL**xx**WE**x**K**xxx**L** motif identified by SPOT alanine scanning (Fig. 3D). The results therefore support that the USP8 Rhod domain binds to two distinct types of motifs (Fig. 4A, B). We used the motifs to scan for matching motifs in previously reported interactors using the SLiMSearch algorithm [39] (Supplementary Table S8 A). As there was no clear match for the TET3-type motif except from only one hit, we focused on the KIAA1614-type motif. We designed an array containing 42 peptides from known interactors, including one peptide from Ubiquitin carboxyl-terminal hydrolase 21 (USP21_156 − 171_), which matched the full Rx[LI]xGxxxPxxL motif, and ligands partially matching the motif: 13 peptides matching Rx[LI]xG, 19 and 8 peptides matching PxxL[LM] or SSPxxL sequences, respectively (Supplementary Table S8B). Most peptides did not bind or resulted in low SPOT intensity (Supplementary Fig. S6; Supplementary Table S8B). The strongest SPOT intensity was found to be the positive control (KIAA1614_568 − 583_), followed by a Rx[LI]xG containing peptide from Keratin-85 (KRT85_41 − 55_), a SSPxxL[LM] containing peptide from the tumour protein 63 (TP63_608 − 622_), and the USP21_156 − 171_ peptide that contains the full motif (Fig. 4C). Both TP63 and USP21 are previously reported USP8 substrates [46, 47], and the interactions may potentially contribute to substrate targeting. Weaker signals were also observed for the SSPxxL containing peptide from the RAB3A interacting protein (RAB3IP_82 − 95_), which has been found to interact with USP8 through affinity proteomics [48], as well as, for two distinct peptides from MCPH1 and SHANK3 (SH3 and multiple ankyrin repeat domains protein 3), both known substrates of USP8 [4, 49]. As the SHANK3 peptides (_351-_SYAKR**R**R**L**A**G**PSGLA_−365_ and _392-_SLRSL**P**HQ**LL**LQRLQ_−406_) are closely located in the primary structure of SHANK3, it is tempting to speculate that both motifs contribute to the interactions between the full-length proteins by increasing the local concentration of binding motifs. Peptide SPOT array alanine scanning of USP21_156 − 171_ peptide clearly confirmed the importance of the arginine at the p1 position of the motif (Rx[LI]xGxxxPxxL[LM]), but other mutations had less distinct effects (Fig. 4D). The SSPxxL[LM] motif used to predict the interaction with the TP63_608 − 622_ peptide was validated by the SPOT array alanine scanning, which also revealed a contribution of the flanking regions (Fig. 4E). Taken together, we find that the USP8 Rhod domain is a peptide binding domain that can be bound by peptides of two distinct types of motifs, with the Rx[LI]xGxxxPxxL motif and variants thereof being found in known USP8 substrates.

## Discussion

In this study, we explored the motif-based interactions of USP8 MIT and Rhod domains through a combination of phage display, alanine-scanning peptide-arrays, deep mutational scanning, AF3 modelling and affinity measurements. For both domains we uncover limited yet diverse sets of ligands. The USP8 MIT domain has previously been reported to bind MIM1 motifs in ESCRT-III proteins [17]. Here, we uncover that it has a preference for a MIM1-variant motif [DE][LIF]x{2,3}R[FYIL]xxL[LV] and also binds to truncated versions of the motif (DFxxR and RFxxL). The results are in agreement with a previous study that based on crystal structures of the MIM1 motif of IST1 in complex with three MIT domains suggested that a LxxRΦxxL (Φ: hydrophobic amino acid) motif is enough for binding to MIT domains [50]. Our study expands the repertoire of MIM1-like motif containing ligands and highlights the plasticity of the motif. Furthermore, we identified a distinct (or highly degenerate) motif in the DBP and EFCAB12 peptides binding to the USP8 MIT domain, expanding the repertoire of different motif classes recognized by the MIT domain.

In the case of the USP8 Rhod domain we uncovered that it is a peptide binding module, and to our knowledge, this is the first time that the Rhod domain has been reported to bind to peptides. Affinity measurements revealed that the KIAA1614_568 − 583_ and the TET3_1705 − 1720_ bind the USP8 Rhod domain with micromolar affinities, and DMS analysis established that the two peptides bind the Rhod domain using distinct motifs. The proposed USP8 Rhod binding Rx[LI]xGxxxPxxL[LM] motif was found to have some predictive power in terms of finding USP8 binding sites in previously reported ligands and substrates. Partial motifs (Rx[LI]xG and PxxL) were also found to be sufficient for binding, although the affinities in several cases appeared to be low based on the relative SPOT array intensities. Based on the occurrence of the Rhod binding motifs in known USP8 substrates (e.g. USP21, TP63 and SHANK3) it is likely that the interactions are of importance for substrate targeting (Fig. 4F), although further analysis will be needed to shed more light into the functional importance of the interactions in a cellular setting.

USP8 has garnered significant interest in biological and clinical research over the past decades due to its association with many diseases, making it a promising therapeutic target [51–53]. This study provides novel insights into the short linear motif-based interactions of USP8 MIT domain and demonstrates that the USP8 Rhod domain is a peptide-binding module that may contribute to substrate targeting (Fig. 4F), information which can be used to gain a better fundamental understanding of its cellular function and potentially can be of use for the development of USP8 specific inhibitors.

## Materials and methods

### Plasmids

The constructs encoding the human USP8 MIT (1-142) and USP8 Rhod (174–317) (UniProt: P40818) domains in the pETM33 vector were synthesized by GenScript. Sequence identities were confirmed by Sanger sequencing.

### Protein expression and purification

The GST-tagged MIT and Rhodanese domains were expressed in *Escherichia coli* (*E. coli*) BL21-Gold (DE3). The proteins were overexpressed in 2YT media (5 g/L NaCl, 16 g/L tryptone and 10 g/L yeast extract) and incubated at 18 °C for 18 h after induction with 0.3 mM isopropyl β-D-1 thiogalactopyranoside (IPTG) at OD_600_ of 0.6–0.8. Bacterial cells were harvested by centrifugation at 4,000 $\:\times\:$*g* at 4 °C. The pellets were resuspended in lysis buffer containing 1x phosphate buffered saline (PBS), 5 mM MgCl_2_, 10 µg/mL lysozyme, cOmplete™ EDTA-free protease inhibitor cocktail (Roche), 10 µg/mL DNaseI and incubated for 1 h shaking at 4 °C. The cells were lysed with 20 s sonication for phage display or using a cell disruptor at 20 kPSI for affinity measurements and SPOT arrays. The cell lysate was pelleted by centrifugation at 16,000 $\:\times\:$*g* at 4 ° for 1 h. The supernatant was incubated with glutathione (GSH) sepharose resin (Cytiva) for 1 h at 4 °C shaking. The resin was washed with 1x PBS pH 8.0 and the GST-tagged protein was eluted with 10 mM reduced glutathione in 50 mM Tris pH 8.0 for phage display. For affinity measurements the GST tag was cleaved on GSH sepharose beads with HRV 3 C Protease in cleavage buffer (50mM Tris pH 7.5, 150mM NaCl, 1mM DTT) overnight at 4° C. The sample was loaded on a gravity column and the cleaved protein was collected in the flow through to separate it from the HRV 3 C Protease and GST-tag. The sample was buffer-exchanged to 50 mM sodium phosphate buffer pH 7.4 with 1mM DTT using a PD-10 desalting column (Cytiva) and concentrated using Amicon^®^ Ultra Centrifugal Filters. The purity and size of the proteins were analyzed by SDS-PAGE electrophoresis.

### DMS library design and construction

The DMS phage library was designed based on four peptide ligands identified as ligands of the USP8 MIT and USP8 Rhod domains through ProP-PD selections (MLPH_481 − 496_: SEVSDIESRIAALRAA, EFCAB12_1 − 16_: MDDDYEAYHSLFLSLL, KIAA1614_568 − 583_: GMERVLGGLSSPLRLL, TET3_1705 − 1720_: GLALWEAKMKQLAERA). The residues of these 16 amino acid-long wild-type peptides were substituted to all natural amino acid residues, except cysteine, in all positions (Supplementary Table S2A). The designed peptide sequences were reverse-translated to oligonucleotides optimized to the codon usage of *E. coli*. The required flanking regions (5´CAGCCTCTTCATCTGGC and 3´GGTGGAGGATCCGGAG) were added for library construction. The library was generated as previously described [28, 54]. The completeness of the library was confirmed by sequencing the *naive* phage library and a 100% coverage was observed at peptide level after NGS analysis, that is, all designed peptides were present in the physical library (Supplementary Table S2A).

### Peptide-phage display selections

Four rounds of phage selections were carried out using the second generation human disorderome library [28] or the constructed deep mutational scanning library. At least three individual replicates were used for each bait protein. 10 µg of GST-tagged proteins or GST control (diluted in PBS from the stock of proteins kept in the purification elution buffer) were immobilized in 100 µL PBS on a 96-well MaxiSorp (Nunc) plate overnight at 4 °C. The plates were incubated with blocking buffer (with 0.5% BSA in PBS) for 1 h at 4 °C followed by washing with 0.05% Tween 20 in PBS four times. The naïve phage library (10^11^ CFU) was diluted in 1/5^th^ volume PEG/NaCl (20% PEG-8000, 0.4 M NaCl) and 1X PBS, incubated on ice for 10 min and centrifuged at 10,000 $\:\times\:$*g* for 10 min. The pellet was resuspended in the volume of PBS (100 µL/ well). The GST-coated control plate was incubated with 100 µL library per well in PBS for 1 h at 4 °C to remove non-specific binders. The supernatant was transferred to the target wells and incubated for 2 h at 4 °C. The wells were washed five times with 0.05% Tween 20 in PBS and the bound phages were eluted with 100 µL per well log-phase *E. coli* OmniMAX by incubating at 37° C while shaking. After 30 min, M13KO7 helper phages were added and incubated for an additional 45 min. The bacterial cultures were transferred to 1 mL 2YT supplemented with 100 µg/mL carbenicillin, 30 µg/mL kanamycin and 0.3 mM IPTG and grew overnight at 37 °C while shaking. The following day the cultures were centrifuged for 10 min at 2,000 $\:\times\:$*g*, 4 °C. 1/10th volume of 10$\:\times\:$ PBS was added to adjust the pH and heated at 65 °C for 10 min. The phage pools from each round were used as in-phages for the next round of selections.

### Phage pool ELISA

For analysing the phage enrichment of each selection round, phage pool ELISA was performed. 10 µg of GST-tagged proteins or GST control were immobilized and blocked with BSA as described for phage display experiments. 100 µL out-phages from each round of selection were incubated with the coated proteins for 1 h at 4 °C and washed four times with 0.05% Tween 20 in PBS. The wells were incubated with 100 µL anti-M13 HRP-conjugated antibody (1:5000) (Nordic Biosite) for 1 h at 4 °C and washed four times with 0.05% Tween 20 in PBS and 1 time with PBS. TMB substrate (Seracare, Cat: 5120-0047) (100 µL/well) was added until a cyan color developed. The reaction was stopped with 0.6 M H_2_SO_4_ (100 µL/well) and the absorbance at 450 nm was measures with SpectraMax iD5 MultiMode Microplate Reader (Molecular Devices).

### Next-generation sequencing (NGS) and data analysis

The samples were prepared for NGS as previously described [28, 54]. Peptide-coding regions of the enriched phage pools were amplified and barcoded using Phusion High-Fidelity PCR Master Mix (Thermo Fisher) for 22 cycles and confirmed by 2% agarose gel electrophoresis. The PCR products were normalized with 25 µL Mag-bind Total Pure NGS beads (Omega Biotek) and 10 µL of each eluted sample (Qiagen Elution Buffer) was pooled and cleaned-up with a PCR purification kit (Qiagen). The PCR product was confirmed by 2% agarose electrophoresis and the band was extracted with QIAquick Gel extraction Kit (Qiagen) and eluted with 30 µL TE buffer. The dsDNA was quantified using QuantiT PicoGreen dsDNA Assay Kit (Thermo Fisher) and sent for NGS (Illumina MiSeq v3, 1 × 150 bp read setup, 20% PhiX, NGI SciLifeLab facility, Sweden). The NGS data were demultiplexed, adapter regions were trimmed and sequences with an average quality of at least 20 were translated to peptide sequences. The NGS data were analysed using custom Python scripts. The total read NGS counts for each selection experiment of each peptide sequence were compiled and annotated in PepTools (https://slim.icr.ac.uk/tools/peptools/) [28]. Since ProP-PD is in essence a competition assay, the possibility of the input phage library influencing the selection outcome was explored. For this, the Pearson correlation coefficient, *p*-value and coefficient of determination were calculated for all peptide NGS count fractions in the library coverage vs. in the selections results (Supplementary Fig. S7; Supplementary Table S2B; Supplementary Table S3B; Supplementary Table S7B). Pearson correlation coefficient and p-value were calculated using Python’s SciPy library [55].

### Position-specific scoring matrix (PSSM) generation for DMS data

A frequency PSSM was generated for each wild type and its corresponding mutant peptides. A single peptide NGS count value was calculated from the sum of the raw counts of replicates and the counts from different selection days for each specific peptide [37]. Counts were then turned into fractions by dividing each one by the total counts for the replicate, and then an average was calculated among the different replicas for each peptide (Supplementary Table S3A; Supplementary Table S7A). The position of each amino acid substitution was identified by comparing each peptide to the wild type peptide. The results were used to generate a PSSM from the peptide NGS counts fractions (Supplementary Table S3C-D; Supplementary Table S7C-D). The frequency PSSM (PSSMfreq) and the DMS score for each substitution was generated by first normalizing all peptide counts fractions in the selection experiments to their fractions in the library coverage, and then converting each column of the PSSM (NGS counts fractions of all 19 substitutions (except cysteine) at a given position of the wild type peptide) to a frequency value by dividing the counts fraction of each amino acid substitution by the sum of the column according to Eq. 1:

$$\:{PSSM}_{{freq}_{\left(c,i\right)}}=\frac{{normalized\:fraction}_{(c,i)}}{{\sum\:}_{i=1}^{19}{normalized\:fraction}_{(c,i)}}$$

An enrichment PSSM (PSSMenr) relative to the wild type frequency was also generated, by calculating the quotient of the wild type and mutant frequencies per column, dividing the highest frequency value by the lowest according to Eq. 2:

$$\:{PSSM}_{enr\left(wt,mut\right)}=\left\{\begin{array}{c}\frac{wt}{mut}\text{,}\text{}\text{i}\text{f}\text{}wt\ge\:mut\\\:\frac{\begin{array}{c}mut\end{array}}{wt}\text{,}\text{}\text{i}\text{f}wt<mut\end{array}\right.$$

Logos were created from the PSSMs by calculating the relative enrichment of each amino acid at each position using the Eq. 3:

$$\:{Relativeenrichment}_{\left(c,i\right)}=\frac{{PSSM}_{{freq}_{\left(c,i\right)}}-expectedfrequency}{expectedfrequency}$$

The expected frequency for any amino acid was 1/19 (excluding cysteines). Logos for the enrichment matrices were plotted using Python’s library Logomaker [56].

### Peptide SPOT array analysis

SPOT array alanine scanning of peptides was designed, and cellulose-bound peptides were synthesized using Fluorenylmethyloxycarbonyl (Fmoc) solid phase synthesis with a MultiPep synthesizer (INTAVIS Peptide Services, Tübingen, Germany). The membranes of predicted peptides were purchased by JPT Peptide Technologies (Berlin, Germany). The membranes were incubated with blocking buffer containing 5% skim milk in TBST (50 mM Tris, 150 mM NaCl pH 7.5, 0.05% Tween-20) for two hours at room temperature. The blocked membranes were incubated with GST-tagged proteins (100 µM for MIT domain and 150 µM for the Rhodanese domain) in blocking buffer overnight at 4 °C. After rinsed with TBST the membranes were incubated with HRP-conjugated anti-GST antibody (Cytiva, RPN1236; 1:3000 dilution) in blocking buffer for 1 h at 4 °C and subsequently rinsed with TBST to remove any excess antibody. The ECL reagent (Clarity Max Western ECL substrate, 1705062, BioRad) was used for chemiluminescence read-out with the ChemiDoc Imaging system (BioRad). The images were analysed with Fiji (ImageJ2 version 2.9.0). SLiMSearch4 was used to design the peptide arrays with known interactors predicted to contain the motifs. The background signal intensity was subtracted, and the corrected signal intensities were normalized to the positive control and presented as percentage.

### Fluorescence polarization affinity measurements

The proteins were buffer exchanged prior to affinity measurements in 50 mM sodium phosphate buffer pH 7.4 supplemented with 1 mM DTT using a PD-10 desalting column (Cytiva) according to the manufacturer’s instructions. The peptides were obtained from GeneCust (France) with > 95% purity. Measurement of the direct binding with a constant concentration of FITC-labeled peptides was performed by increasing concentration of the protein. The K_D_ value from the direct binding experiment was obtained by fitting against a quadratic equation (Eq. 4) where “pept” indicates the fixed probe peptide concentrations, X indicates the protein concentration, the constant A is the signal amplitude divided by probe peptide concentration, and B is the plateau value. Eq. 4:

$$\:Y=\frac{A*pept+X+{K}_{D}+\sqrt{\left(pept+X+{K}_{D}\right)-4pept*X}}{2}+B$$

To determine the K_D_ of the unlabeled peptides, 1.5 µM of protein was preincubated with 10 nM FITC-labeled peptide to form a complex. Displacement experiments were carried out by increasing concentration of the unlabeled peptides. The data were fitted with a sigmoidal dose-response equation. The results were analysed with GraphPad Prism version 9.2.0.

### Docking

Docking of the peptides was performed with AlphaFold2-multimer-v3 [42] using a local installation of ColabFold [57], with default parameters, or with the AlphaFold3 webserver, and the models selected for visualization were selected based on ipTM values and manual inspection. All structure visualizations were created with ChimeraX v1.8.

## Electronic supplementary material

Below is the link to the electronic supplementary material.

Supplementary Material 1

Supplementary Material 2

Supplementary Material 3

Supplementary Material 4

Supplementary Material 5

Supplementary Material 6

Supplementary Material 7

Supplementary Material 8

Supplementary Material 9

## Data Availability

The protein interactions will be submitted to the IMEx (http://www.imexconsortium.org) consortium. ProP-PD results will also be made available through the ProP-PD portal.
