# Supplementary material for: Elucidation of short linear motif-based interactions of the MIT and rhodanese domains of the ubiquitin-specific protease 8
Source: Biol Direct. 2025 May 6;20:59. doi: 10.1186/s13062-025-00638-7 (PMC12057046; doi:10.1186/s13062-025-00638-7)
Supplement: Supplementary file 1 — Supplementary Material 1 [file 13062_2025_638_MOESM1_ESM.pdf]

Elucidation of short linear motif-based interactions of the MIT and rhodanese domains of the ubiquitin-specific protease 8

Aimiliani Konstantinou[1], Julia K. Varga[2], Alicia Córdova-Pérez[3], Leandro Simonetti[1], Lidia Gomez-Lucas[1], Ora Schueler-Furman[2], Norman E. Davey[4], Yogesh Kulathu[3] & Ylva Ivarsson[1]

Affiliations

1. Department of Chemistry-BMC, Uppsala University, Box 576, 751 23 Uppsala, Sweden
2. Department of Microbiology and Molecular Genetics, Institute for Biomedical Research IMRIC, Faculty of Medicine, Hebrew University of Jerusalem, Israel
3. MRC Protein Phosphorylation & Ubiquitylation Unit, School of Life Sciences, University of Dundee, United Kingdom
4. Division of Cancer Biology, The Institute of Cancer Research (ICR), United Kingdom

Supplementary figures

Supplementary Fig. 1. AlphaFold3 (AF3) model of full-length USP8.

Supplementary Fig. 2. SPOT array alanine scanning of SETD1B$_{492-507}$ peptide binding to the MIT domain.

Supplementary Fig. 3. AF3 modeling of superimposed peptides with MIM1 motif variation.

Supplementary Fig. 4. USP8-MIT SPOT array of predicted ligands from known USP8 interactors containing motif variations generated by DMS.

Supplementary Fig. 5. Crystal structure of CDC25B catalytic domain (PDB id 1qb0) superimposed to AF3 model of USP8 Rhod bound to TET3$_{1705-1720}$.

Supplementary Fig. 6. USP8-Rhod SPOT array of predicted ligands from known USP8 interactors containing motif variations generated by DMS.

Supplementary Fig. 7. Evaluation of the effect of peptide counts in the naïve USP8_DMS phage library on selection results.

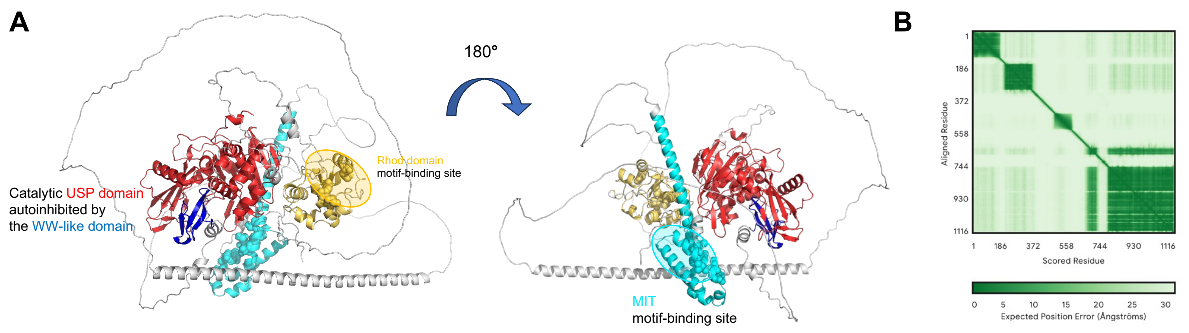

Supplementary Fig. 1. Cartoon representation of the AF3 model of full-length USP8 (PyMol). (**A**) The N-terminal MIT domain is shown in cyan, the catalytically inactive Rhod domain is shown in red, the autoinhibitory WW-like domain is shown in blue and the C-terminal catalytic domain is in red. Other parts of the protein are in grey. The proposed motif-binding sites in the Rhod domain and the MIT domain are highlighted. (**B**) The expected position error of the model as provided by the AF3 server.

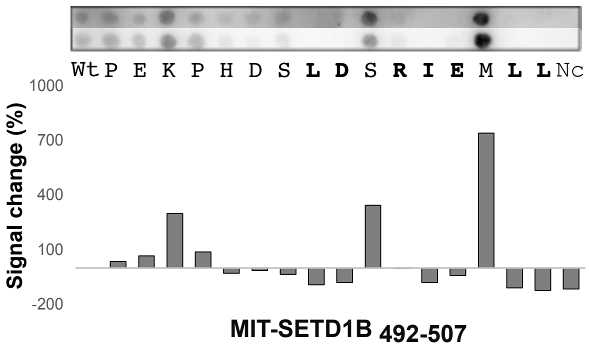

Supplementary Fig. 2. Peptide SPOT array alanine scanning of SETD1B$_{492\text{-}507}$ peptide binding to the MIT domain. Amino acid residues disrupting binding when mutated to alanine are shown in bold. Signal intensities were normalized to wild type (Wt) and displayed as average percent signal change; Nc: negative control (scrambled sequence).

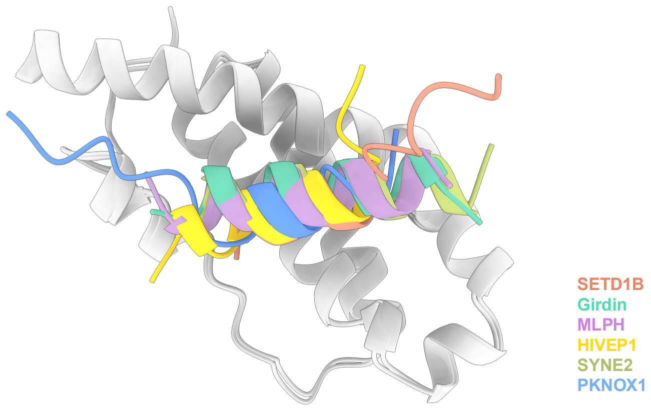

Supplementary Fig. 3. Superimposed AF3 models of peptides with MIM1 motif variation. The peptides are predicted to bind to the same USP8 MIT domain binding pocket.

A

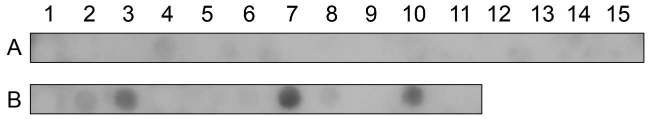

B

Position	GeneName	Predicted Motif	Percentage (%)
A1	BIRC6	L.LL	12
A2	CD83	L.LL	6
A3	EGFR	L.LL	6
A4	FZD5	L.LL	24
A5	HIF1A	L.LL	0
A6	NOTCH1	L.LL	2
A7	NR3C1	L.LL	-4
A8	PDGFRB	L.LL	-3
A9	PTPN23	L.LL	-7
A10	RNF149	L.LL	3
A11	SHANK1	L.LL	-6
A12	TGOLN2	L.LL	-5
A13	TMEM79	L.LL	-4
A14	TP63	L.LL	8
A15	USP28	L.LL	1
B1	KIF23	LE..[IFY]..L	7
B2	CFLAR	RL	31
B3	CDH1	RF..L	71
B4	FGFR2	RF..L	3
B5	IST1	RF..L	5
B6	SHANK1	RF..L	7
B7	DNAJB6	DFx{2,3}R	100
B8	VIRMA	F...[RK]	7
B9	CHMP2A	D..F.R	-8
B10	DBP Positive control		67
B11	SYNE2 Negative control		-4

Supplementary Fig. 4. USP8-MIT SPOT array of predicted ligands from known USP8 interactors containing motif variations generated by DMS. (A) SPOT array of predicted peptides found in SLiMSearch to contain matches of the motif and are previously reported interactors of USP8. **(B)** Peptides with the motif match and the percentage normalized to the highest intensity and corrected for background. Additional information about the peptides is provided in Supplementary Table 4B.

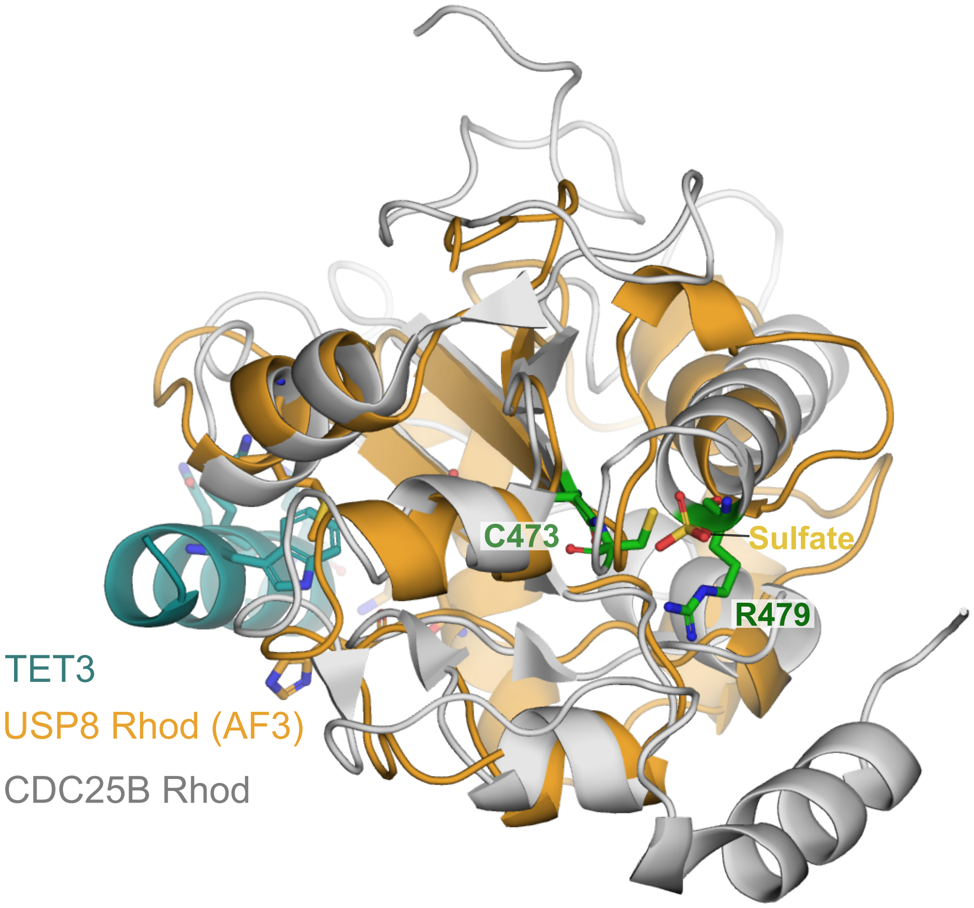

Supplementary Fig. 5. Crystal structure of CDC25B catalytic domain (1qb0) superimposed to AF3 model of USP8 Rhod bound to TET3$_{1705\text{-}1720}$. The catalytic C473 and R479 of CDC25B are indicated.

A

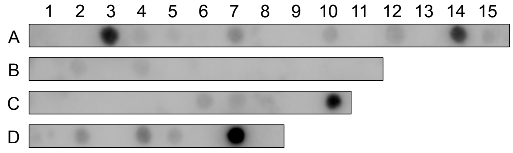

B

Position	GeneName	Predicted Motif	Percentage (%)
A1	BIRC6	R.[LI].G	5
A2	EEA1	R.[LI].G	2
A3	KRT85	R.[LI].G	52
A4	KRT85	R.[LI].G	5
A5	MAGEL2	R.[LI].G	2
A6	MAGEL2	R.[LI].G	0
A7	MCPH1	R.[LI].G	11
A8	MCPH1	R.[LI].G	-2
A9	PJA1	R.[LI].G	-1
A10	SHANK1	R.[LI].G	5
A11	SHANK1	R.[LI].G	-2
A12	SHANK3	R.[LI].G	7
A13	SHANK3	R.[LI].G	-1
A14	USP21	R.[IL].G...P..L	39
A15	KIF23	WE.K	6
B1	BACE1	P..L[LM]	1
B2	DNAJB6	P..L[LM]	5
B3	FZD5	P..L[LM]	1
B4	MAGEL2	P..L[LM]	4
B5	MAGEL2	P..L[LM]	-1
B6	MAGEL2	P..L[LM]	-1
B7	MAGEL2	P..L[LM]	-1
B8	MAGEL2	P..L[LM]	-1
B9	MAGEL2	P..L[LM]	-2
B10	MAGEL2	P..L[LM]	-1
B11	NBR1	P..L[LM]	-1

Position	GeneName	Predicted Motif	Percentage (%)
C1	NTRK1	P..L[LM]	1
C2	PTPN23	P..L[LM]	0
C3	SHANK1	P..L[LM]	-1
C4	SHANK1	P..L[LM]	-1
C5	SHANK1	P..L[LM]	-1
C6	SHANK1	P..L[LM]	7
C7	SHANK3	P..L[LM]	7
C8	STAT6	P..L[LM]	4
C9	STAT6	SSP..L[LM]	0
C10	TP63	SSP..L[LM]	45
D1	EPS15	SSP..L	2
D2	MCPH1	SSP..L	9
D3	PTPN23	SSP..L	-1
D4	RAB3IP	SSP..L	17
D5	RASGRF1	SSP..L	6
D6	TMEM79	SSP..L	1
D7	KIAA1614 Positive control		100
D8	Random KIAA1614 Negative control		4

Supplementary Fig. 6. USP8-Rhod SPOT array of predicted ligands from known USP8 interactors containing motif variations generated by DMS. (A) SPOT array of predicted peptides found in SLiMSearch to contain matches of the motif and are previously reported interactors of USP8. **(B)** Peptides with the motif match and the percentage normalized to the highest intensity and corrected for background. Additional information about the peptides is provided in Supplementary Table 8B.

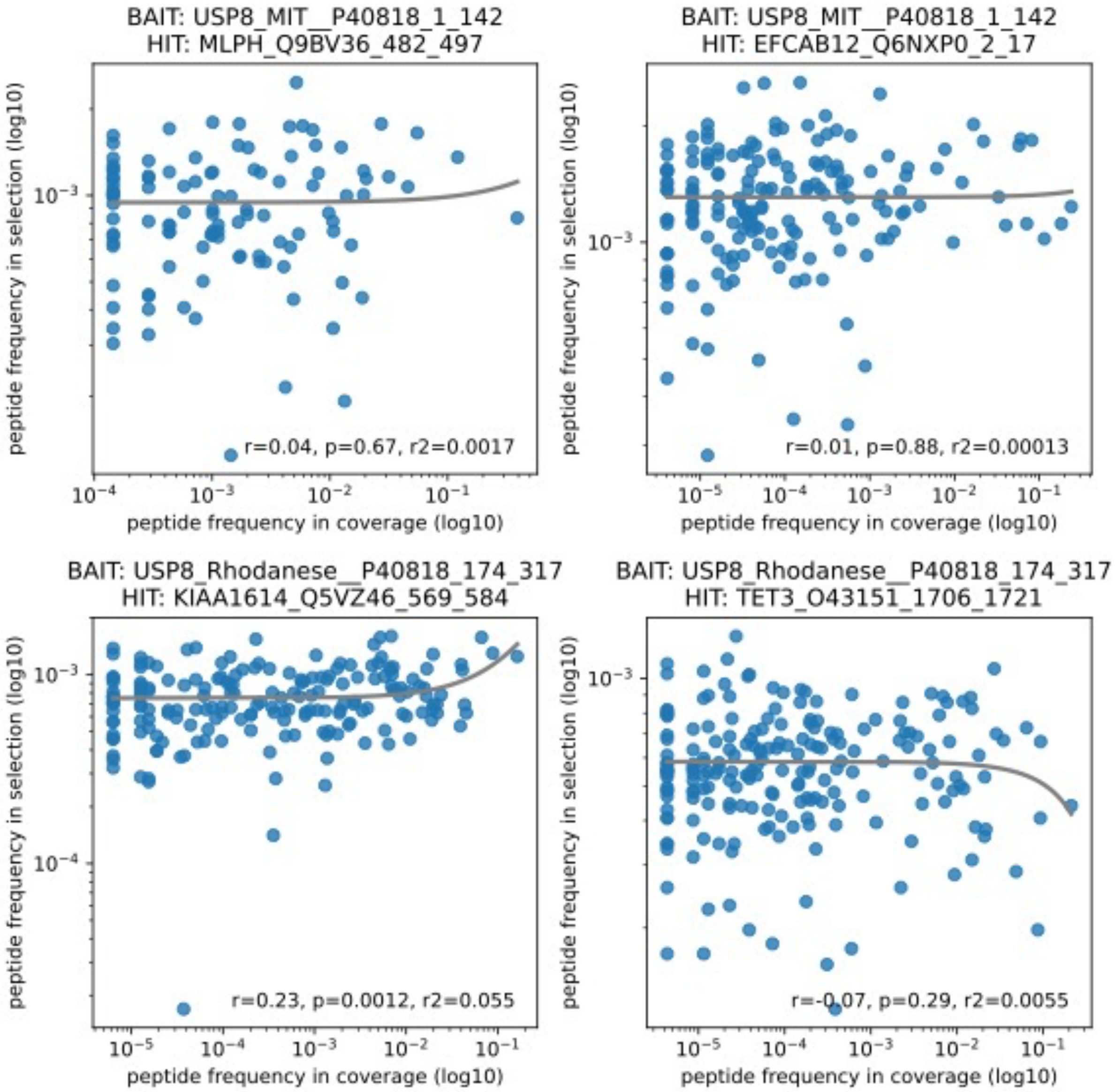

Supplementary Fig. 7. Evaluation of the effect of peptide counts in the naïve USP8_DMS phage library on selection results. Scatter plots with linear regressions for all peptide counts frequencies in the sequenced naive library (coverage) vs. in all selection results are shown, together with their Pearson correlation coefficient (r), p-values (p) and coefficients of determination (r2). A correlation was observed only for the USP8 Rhod and the KIAA1614 peptide (r=0.23 with a p-value < 0.01), for which only 5.5% (r2) of the variability observed in the sequencing counts can be explained by the counts in the coverage.
